# Activity of IL-12/15/18 primed natural killer cells against hepatocellular carcinoma

**DOI:** 10.1007/s12072-018-9909-3

**Published:** 2018-11-22

**Authors:** Lihui Zhuang, Rebecca J. Fulton, Pauline Rettman, A. Emre Sayan, Jonathan Coad, Aymen Al-Shamkhani, Salim I. Khakoo

**Affiliations:** Faculty of Medicine, Southampton General Hospital, University of Southampton, Tremona Road, Southampton, SO16 6YD UK

**Keywords:** Innate immunity, Immunotherapy, Cytokines, Liver, Cancer

## Abstract

**Background:**

Hepatocellular carcinoma (HCC) is common, but remains difficult to treat. Natural killer (NK) cells are cells of the innate immune system that have potent anti-cancer activity. Recent work has shown that stimulation with IL-12/15/18 leads to the generation of NK cells with enhanced functional and putative “memory” properties. We have investigated the activity of these NK cells against HCC cell lines in vitro and in a mouse model.

**Methods:**

NK cells from healthy donors or individuals with HCC were activated with IL-12/15/18 in vitro and tested for cytotoxic activity against a panel of human HCC cell lines. IL-12/15/18 primed murine NK cells were then infused into a murine model of spontaneously arising HCC to test for anti-tumor activity.

**Results:**

NK cells from patients and healthy controls had similar expression levels of activating and inhibitory NK cell receptors. However, proliferation of NK cells from HCC patients was weaker than healthy controls in response to IL-12/15/18 and IL-2 (*p* < 0.001 at day 9). In vitro, NK cells from both groups of individuals killed HCC targets to similar levels and this was unrelated to NKG2D expression. In a spontaneous model of HCC, IL-12/15/18 activated NK cells trafficked to the liver and resulted in lower levels of spontaneous HCC formation (*p* < 0.01).

**Conclusion:**

Cytokine-primed NK cells from patients with HCC have similar levels of activity against HCC cell lines as those from healthy controls. This type of activated NK cell has immunotherapeutic potential against hepatocellular carcinoma.

**Electronic supplementary material:**

The online version of this article (10.1007/s12072-018-9909-3) contains supplementary material, which is available to authorized users.

## Background

Hepatocellular carcinoma (HCC) is a common complication of end-stage liver disease and develops on a background of liver fibrosis as a consequence of most causes of chronic liver disease, including alcoholic liver disease, non-alcoholic fatty liver disease and chronic viral hepatitis. It is the sixth most common type of cancer worldwide and it is the third most common cause of cancer death. However, the treatment for HCC is limited and if transplantation or resection is not possible, the prognosis is poor. Novel therapies are, therefore, urgently required.

Natural killer (NK) cells are important components of the anti-tumor immune response. NK cells express an array of receptors, including inhibitory, activating, adhesion and cytokine receptors. The integration of signals from these receptors determines whether or not NK cell becomes activated, which in turn enables them to detect target cells while sparing healthy cells. In certain conditions, such as viral infections or cancer, cells are stressed and up-regulate the ligands for activating receptors on NK cells, thus triggering NK cell activation [1]. Therefore, NK cells have a strong immunotherapeutic potential but the difficulty of generating large number of NK cells that are safe and fully functional in vivo limits their clinical utility.

The immunotherapeutic importance of NK has best been shown in haplo-identical bone marrow transplantation in which mismatched NK cells exert a key role in killing residual leukemic blasts without causing graft-versus-host disease [2]. However, the exploitation of NK cells in solid tumor therapy is still limited and unsatisfactory. Previous work has demonstrated that in HCC patients there was significant reduction in NK cell number both in peripheral blood and liver [3]. In vitro both hepatic and peripheral NK cells from HCC patients had significantly diminished cytotoxic ability against K562 cells, compared to healthy donors. However, studies have shown that the expression of the ligands for the activating NK cell receptor NKG2D in HCC tissue correlates with outcome [4]. Another genome-wide association study identified a susceptibility locus in the 5′ flanking region of MICA for HCV-induced hepatocellular carcinoma [5]. Thus, the NKG2D:NKG2D ligand axis could be a potential therapeutic target in HCC.

Immunotherapy represents an important potential addition to the treatment of HCC. Despite a number of studies showing that NK cells influence the outcome of HCC, their utility as treatment for HCC remains unknown. There are a number of potential mechanisms for activating NK cells and IL-2 still remains the mainstay. However, although IL-2 can activate NK cells in vitro, there may be limited NK cell expansion [6, 7]. Further attempts to expand purified NK cells by culture in IL-2 for 12 days demonstrated a less than 10-fold expansion of NK cells [8]. Culture of purified CD56 + CD3-NK cells in IL-2 and IL-15 for 14 days gave a 34.9 ± 10.4 fold expansion [9]. Cell-surface receptors on NK cells have also been targeted to enhance proliferation. For instance, 4-1BB is a member of the TNF receptor superfamily that is expressed on T cells and NK cells following activation [10]. Antibodies to 4-1BB are currently undergoing clinical development [11]. In the context of adoptive NK cell therapy, a leukemia cell line, K562, was modified to express a membrane-bound form of IL-15 and 4-1BB (CD137) ligand to enhance NK cell expansion [12]. After irradiation, this cell line activated and induced a median 21.6-fold expansion of CD56 + CD3- NK cells from peripheral blood after 7 days, which was an improvement to using IL-15 or 4-1BB ligand alone [12, 13].

Recent work has described a cytokine cocktail of IL-12/15/18 to pre-activate mouse NK cells, and then following infusion, these NK cells proliferate and kill tumor cells in vivo [14]. This may also be applicable to human NK cells and has been shown to generate a putative “memory” NK cell phenotype with enhanced functionality [15, 16]. A recent phase I study of these cells demonstrated efficacy in refractory acute myeloid leukemia [17]. The aim of this current study was to test the potential for these cytokine-induced NK cells to be anti-HCC agents.

## Materials and methods

### Human NK cell isolation

Peripheral blood mononuclear cells (PBMC) were isolated by Ficoll-paque Premium (GE healthcare, VWR, Lutterworth, UK) and followed the recommended protocol by the manufacturer. Human NK cells were isolated by negative selection using the Miltneyi NK cell Isolation Kit (LS) for “untouched” NK cells according to the manufacturer’s protocol (Miltenyi Biotec, Surrey, UK). Purity was > 85% with less than 1% CD3 + T cells (Supplementary Figure 1).

### Patients

Twenty patients were recruited in total, 15 male and 5 female. The mean age of patients was 71 (66–78), and 9 had alcoholic liver disease, 7 NAFLD and the remainder had a variety of other liver diseases. According to the Barcelona Clinic Liver Cancer (BCLC) staging, patients were classified as follows: one stage 0, two stage A, fourteen stage B, two stage C and one stage D. At the time of recruitment none had been treated for HCC. These were significantly older than healthy controls mean age 31.2 years (20–40), *p* < 0.001.

### In vitro CFSE proliferation assay

NK cells were pelleted and resuspended in 37 °C 5 µM CFSE (Molecular Probes, Invitrogen, Paisley, UK) dissolved in PBS/0.1% BSA at 10 × 10^6^ cells/mL, then incubated at 37 °C for 10 min. Following quenching with five volumes of ice-cold culture media, cells were pelleted and washed. Cells were then seeded in hNK culture medium (RPMI 1640 with 1% L-Glu/P/S and 10% human AB serum) at 1 × 10^6^ cells/mL, 0.2 mL/well, in 96-well U-bottom plate. Cells were cultured with different stimulations: (1) 1000 U/mL IL-2; (2) 10 ng/mL IL-15; (3) 10 ng/mL IL-12, 20 ng/mL IL-15, 100 ng/mL IL-18 overnight, then 100 U/mL IL-2 every 2 days. Cytokines were supplemented every 2 days. CFSE division was checked by flow cytometry, and cell number was quantified using a haemocytometer. For anti-CD137 experiments, an in-house anti-human 4-1BB (SAP, clone 3.28) was diluted in 0.05 M carbonate-bicarbonate buffer (pH 9.6) and added to a 96-well flat-bottom plate at 100 µL/well at 4 °C overnight [18]. The liquid was removed and then NK cells added, and incubated for 1 h at room temperature before the proliferation assay.

### Flow cytometry staining

Cells were washed with washing buffer (1% BSA and 0.1% NaN_3_ in PBS) before blocking with 10% human serum in washing buffer at 4 °C for 30 min. Cells were then stained with antibodies for 30 min at 4 °C before washed for analysis. Anti-human CD3-PerCP (UCHT1), CD56-Alexa 488 (HCD56), NKG2D-APC (1D11), CD158a-FITC (HP-MA4) (Biolegend, London, UK), CD27-APC (M-T271), CD137-PE (4B4-1), IFNγ-PE (B27), Perforin-PE (δG9), CD158b-FITC (CH-L) (BD Pharmingen, England), and NKp46-APC (9E2) (Miltenyi Biotech, Surrey, UK), NKG2A (CD159a)-PE (Z199) (Beckman Coulter) were used. Anti-mouse CD27-APC (LG.3A10), CD137-PE (1AH2) (BD Bioscience, Oxford, UK) was used. Flow cytometric analysis was performed with an Accuri C6 (BD) and data were analyzed using CFlow software (BD).

### Flow cytometry cytotoxicity assay

221, SNU387, SNU398, SNU423, SNU475 cells were cultured in R10 medium (RPMI1640 with 1% Pen/Strep, 1% L-Glu and 10% FBS [all Invitrogen, Life Technologies, Renfrew, UK]) [19]. Huh7, HepG2, PLC cells were cultured in D10 medium (DMEM high glucose no glutamine with 1% Pen/Strep, 1% L-Glu and 10% FBS) [20]. Isolated human NK cells were cultured in hNK medium (R10 + 5% human AB serum (Sigma, Dorset, UK). Target cells were labeled with 10 µM DDAO-SE (Molecular Probes, Invitrogen) and incubated overnight at 37 °C. NK cells were added to target cells and incubated at 37 °C for 4 h. Cells were harvested by Trypsin–EDTA (Invitrogen, Life Technologies) and stained with Live/Dead Fixable Green cell stain (Molecular Probes, Invitrogen). The percentage cytotoxicity was measured by calculating the percentage of dead cells in the target cell population minus the background of dead cells without effectors.

### Murine tumor model

TGFα and c-Myc mice were a kind gift from Dr A Piiper (Goethe-University Frankfurt, Germany) and double transgenic c-Myc/TGFα mice were generated by crossing these mice as previously described [21]. Hepatocarcinogenesis was triggered in male c-Myc/TGFα mice, by the addition of ZnCl_2_ to the drinking water on weaning. Following killing of the mice, livers were weighed, dissected and the number of macroscopically visible tumors counted.

NK cells were isolated from homogenized murine spleens using Miltenyi NK cell isolation kit II for “untouched” NK cells. Purity was > 80% with less than 3% CD3 + T cells (Supplementary Figure 1). Cells were incubated overnight with 10 ng/mL IL-12 (Peprotech, UK), 10 ng/mL IL-15 (R&D, Abington, UK), and 50 ng/mL IL-18 (Caltag-Medsystems, Buckingham, UK) at 37 °C. Cells were harvested, resuspended in PBS + 0.1% BSA and stained with the CellTrace™ CFSE Cell Proliferation Kit. For both the localization and therapeutic studies, 1 × 10^6^ cells were injected intravenously via the tail vein. NK cells were isolated from the liver by homogenizing the liver or spleens. Cells were then pelleted and mononuclear cells isolated for flow cytometry using Ficoll density gradient centrifugation. Cells were stained with anti-CD3-PERCP and anti-NK1.1 (Biolegend) prior to flow cytometry analysis.

### Statistics

Data were analyzed using GraphPad Prism™, using Mann–Whitney, Student’s *T* or Kruskal–Wallis tests where appropriate.

## Results

### HCC cell lines are killed with varying efficacy by NK cells and express different NKG2D ligands

**Fig. 1:**
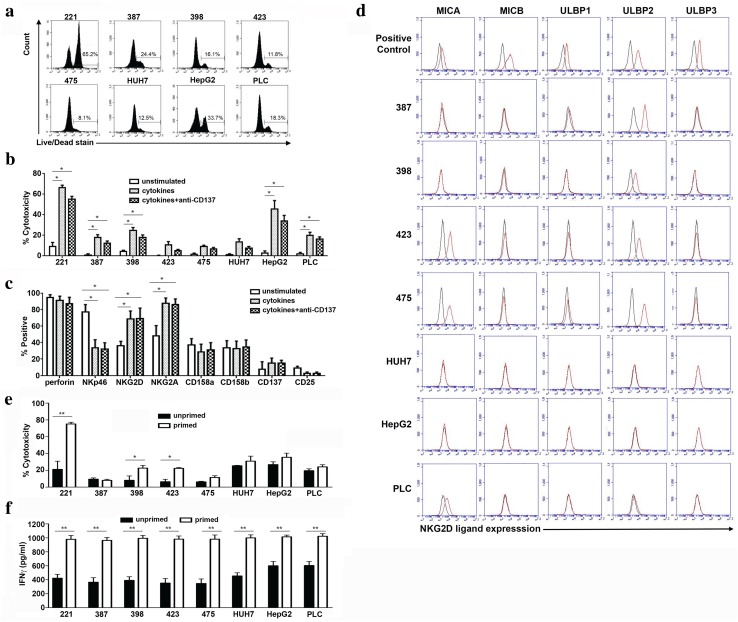
Cytotoxic activity of IL-12/15/18 activated NK cells. **a**–**c** 2 × 10^5^ purified NK cells were stimulated overnight in a 96 well plate with IL-12 (10 ng/ml), IL-15 (20 ng/ml) and IL-18 (100 ng/ml) and IL-2 (100 iu/ml) added on alternate days and then assayed on day 8. For anti-CD137 experiments, plates were pre-coated with anti-CD137 at a concentration of 10 μg/ml. **a**, **b** Cytotoxicity of IL-12/15/18 and IL-2-activated NK cells from healthy controls before and after cytokine stimulation. NK cells were tested against control 721.221 (221) cells and 7 different human liver cancer cell lines, SNU387, SNU398, SNU423, SNU475, Huh7, HepG2, PLC. One representative cytotoxicity assay is shown in **a** and the means and SEM from six donors are shown in **b**. All experiments were performed at an effector:target ratio of 2:1. **c** Expression of receptors on NK cells before and after stimulation with the cytokine cocktail (*n* = 5). For **b** and **c** significance was tested using the non-parametric Kruskal–Wallis one-way ANOVA test (Graph Pad Prism™). Tests were performed individually for each cell line tested. *p* values where shown compare unstimulated cells with cells stimulated either with cytokines alone, or with cytokines plus anti-CD137. For all panels *p < 0.05. **d** Expression of NKG2D ligands on the HCC cell lines by flow cytometry. NKG2D ligands are shown by red lines and isotype control by black lines. Positive control cells lines were K562 (MICA, MICB), Jurkat (ULBP1), HEK293T (ULBP2, ULBP3). Comparison of cytotoxicity (**e**) and IFNγ secretion (**f**) of cultured IL-12/15/18 primed NK cells and unprimed NK cells following incubation with the indicated cell lines for four hours (*n* = 6 donors). Primed NK cells were stimulated as for **a**–**c** and unprimed NK cells were cultured in 100 iu/ml IL-2 on day 0, and subsequently alternate days. Cells were tested at day 8 of culture and experiments were performed at an effector:target ratio of 2:1. Means and SEMs are shown. A paired *t* test was performed for each cell line comparing paired, primed and unprimed, NK cells from each donor (Graph Pad Prism™). For **e** and **f** *p < 0.05, **p < 0.001

To test the potential for IL-12/15/18 cytokine-activated NK cells in HCC immunotherapy, we tested a panel of liver tumor cell lines that represent HCC at a variety of stages of differentiation. NK cells were cultured with the cytokine cocktail plus IL-2 and tested for their killing activity against the HCC lines. Activation of NK cells was associated with an increase in killing for all the cell lines tested (Fig. 1a, b). As CD137 stimulation has been described to enhance NK cell activity in vitro, we also tested the effect of plate-bound anti-CD137 on HCC cell line killing. However, no enhanced effect of CD137 was observed (Fig. 1b and Supplementary Table 1a).

We observed variability in the levels of killing of these cell lines ranging from 9.1% for SNU475 to 45% for HepG2 at E:T ratios of 2:1, which did not correlate well with the stage of cellular differentiation, as Huh7, HepG2 and PLC are well-differentiated cell lines while SNU387, SNU398, SNU423 and SNU475 are poorly differentiated [19]. To correlate this activity with NK cell receptor expression, we stained the NK cells for NKp46 and NKG2D (both activating receptors); CD158a and CD158b (both inhibitory receptors), and CD27 and CD137 (activating TNFR superfamily members) (Fig. 1c and Supplementary Table 1b). We observed up-regulation of NKG2D and NKG2A, but not of NKp46 on cytokine-activated NK cells, and also expression of CD137 on only approximately 20% of NK cells, which may contribute to the lack of efficacy of anti-CD137.

As we observed up-regulation of NKG2D, we next tested the expression of NKG2D ligands (MICA, MICB, ULBP1, ULBP2 and ULBP3) on all the HCC panel cell lines. Although there was variation in expression of NKG2D ligands on these cells, this did not correlate well with susceptibility to NK cell killing (Fig. 1d, Table 1). For instance, neither Huh7 nor HepG2 expressed NKG2D ligands, but HepG2 was killed well (~ 40% specific cytotoxicity) by NK cells, and Huh7 cells were killed to approximately half these levels. Thus, killing of different HCC cell lines is multi-factorial and not specifically associated with the NKG2D:NKG2D ligand axis.

**Table 1:** Expression of NKG2D ligands on human HCC cell lines

	Killing > 10%	MICA	MICB	ULBP1	ULBP2	ULBP3
SNU387	Yes	−	−	−	+	−
SNU398	Yes	−	−	−	+	−
SNU423	No	+	−	−	+	−
SNU475	No	+	−	−	+	−
HUH7	No	−	−	−	−	−
HEPG2	Yes	−	−	−	−	−
PLC/PRF/5	Yes	+	−	−	+/−	−

We next compared the effects of cytokine priming with IL-12/15/18 on NK cell activity against these cell lines. At day 8, we observed higher levels of cytotoxicity by the cytokine-primed NK cells against two HCC cell lines (SNU398 and SNU423) and also the control class I-negative 721 cell line, but similar levels for other cell lines (Fig. 1e and Supplementary Table 1c). Conversely, IFNγ secretion was generally higher with the IL-12/15/18 primed NK cells, consistent with the reported “memory” phenotype of these cells (Fig. 1f, Supplementary Table 1d) [16]. This may represent a generalized enhancement of IFNγ secretion by the cytokine priming, rather than a cell line-specific effect.

### NK cells from patients with HCC proliferate more weakly to cytokines but have equivalent cytotoxic potential

**Fig. 2:**
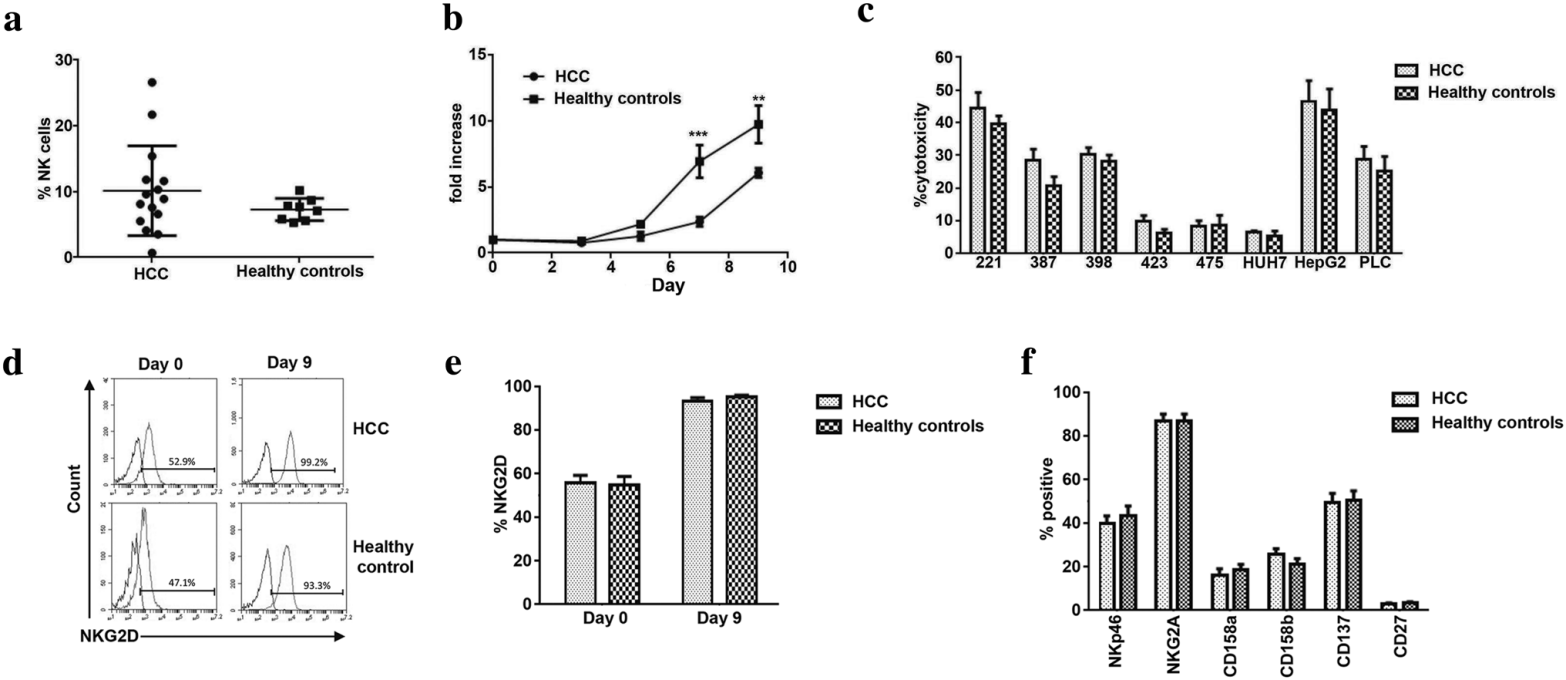
Comparison of activity and markers NK cells from HCC patients and healthy controls. **a** The frequency of CD3-CD56 + NK cells in the peripheral blood from individuals with HCC as compared to healthy controls. **b** NK cells were purified from frozen and thawed PBMC from eight healthy controls and eleven HCC patients and then tested for proliferation using IL-12 (10 ng/ml), IL-15 (20 ng/ml) and IL-18 (100 ng/ml) and IL-2 (100 iu/ml) on alternate days. The fold increase in NK cell number is shown (***p < 0.001 and **p < 0.01, Mann–Whitney test comparing HCC patients and healthy controls using Graph Pad Prism™). **c** Cytotoxicity assays of IL-12/15/18 + IL-2-activated NK cells (day 9) against control 721.221 (221) cells and a panel of HCC cell lines. Assays were performed at an effector:target ratio of 2:1. The means and SEMs of cytotoxicity assays from the eight healthy controls and eleven HCC patients are shown. **d**–**f** Comparison of cell-surface markers on cytokine-primed NK cells from patients with HCC (*n* = 12) and healthy controls (*n* = 7). Representative NKG2D expression from two individuals is shown in **d** and the mean NKG2D expression is shown in **e**. Expression of other markers at day 9 following cytokine stimulation is shown in **f**

To investigate the potential for using autologous NK cells from HCC patients in immunotherapeutic regimes, we studied fifteen individuals with HCC. Patients with HCC had similar number of CD3-CD56 + NK cells as healthy controls, but with a wide range 0.7% to 26.6% in HCC versus 5.3–10.2% in healthy controls (Fig. 2a). NK cells from HCC patients responded to the cocktail of cytokines, but proliferated less well (6 fold versus 10 fold by day 9, *p* < 0.001) (Fig. 2b, Supplementary Figure 2 and Supplementary Table 1e). NK cells from HCC patients did, however, kill targets to similar levels as NK cells from healthy controls, with a similar hierarchy of killing (Fig. 2c and Supplementary Table 1f). Expression of NKG2D was upregulated to similar levels as healthy controls, as was the ability to up-regulate NKG2D under the influence of IL-12/15/18 + IL-2 (Fig. 2d, e, and Supplementary Table 1g). Furthermore, at day 9 we did not detect any significant differences between HCC and healthy controls with any of the markers tested (Fig. 2f and Supplementary Table 1h).

### IL-12/15/18 primed NK cells localize to the murine liver and are associated with lower levels of tumor development

**Fig. 3:**
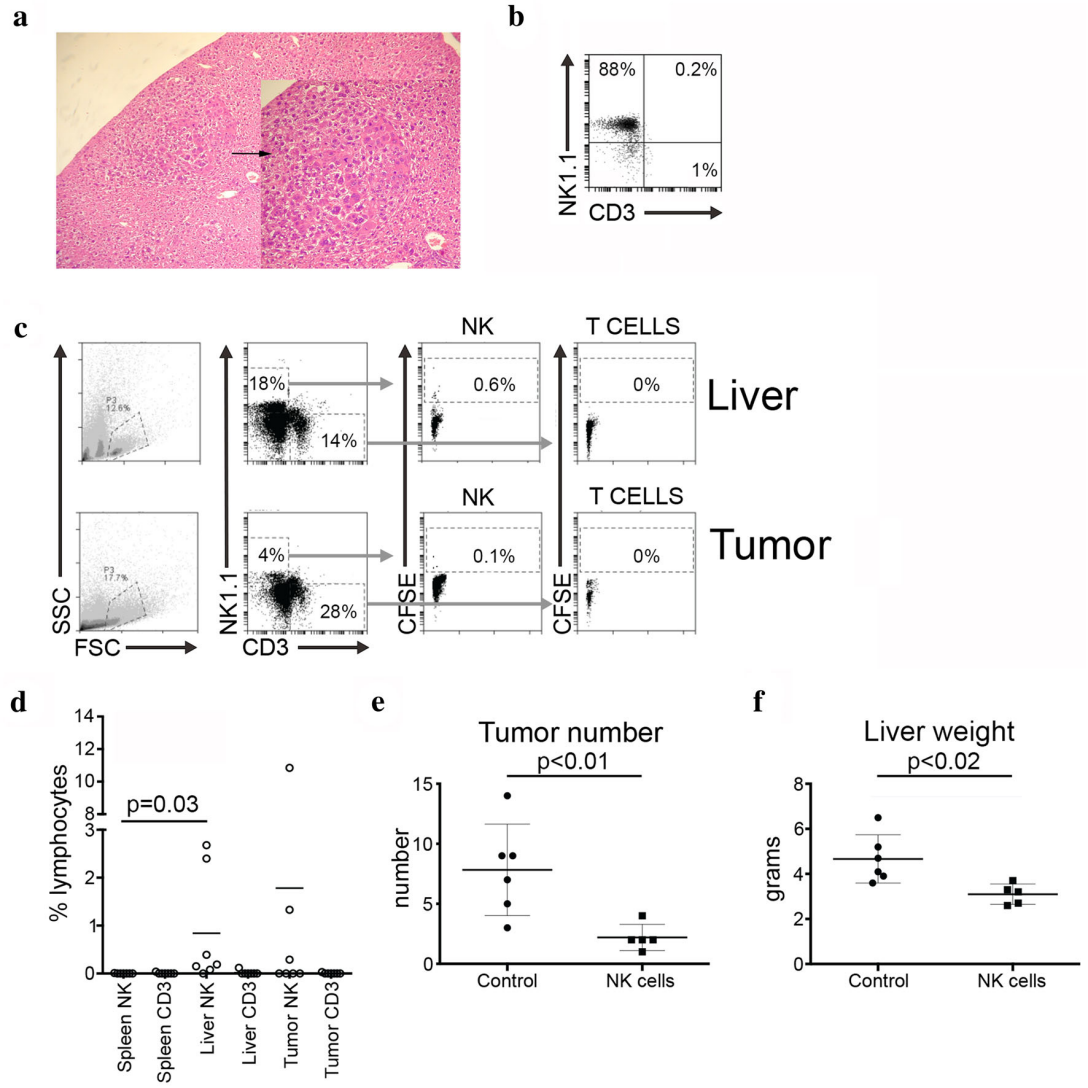
Cytokine-activated NK cells localize to the liver and are associated with a reduced tumor burden. **a** Spontaneous HCC arising in a c-Myc/TGFα double transgenic mouse at low (×10) and higher (×40) power (inset). The HCC is arrowed. **b** Flow cytometry plot of purified NK cells prior to injection into c-myc/TGFα mice. **c**, **d** NK cells were labeled with CFSE and adoptively transferred into tumor-bearing mice. **c** Representative flow cytometry plots indicating gating strategy and CFSE expression on CD3-NK1.1 + NK cells and CD3 + NK1.1- T cells. **d** Frequency of CFSE expressing NK and CD3 + NK1.1-T cells in liver parenchyma, liver tumor and spleen at 24 h post-NK cell infusion. Comparison of spleen and liver NK cells was made using Wilcoxon matched-pairs signed rank test. Comparison of tumor number (**e**) and liver weight (**f**) in mice receiving NK cell infusions compared to PBS controls. Comparison between the two groups of mice was made using Student’s *t* test (Graph Pad Prism™)

Having shown in vitro that cytokine-primed NK cells had activity against HCC cell lines, we next wished to test if they would also localize to the liver and have in vivo activity. We used the c-Myc/TGFα mouse model of spontaneous HCC (Fig. 3a). NK cells were purified from the spleens of TGFα mice, stimulated overnight with IL-12/15/18, labeled with CFSE and then 1 × 10^6^ NK cells were injected via the tail veins of the c-Myc/TGFα mice. NK cells were typically ~ 90% pure with less than 2% CD3 + T cells (Fig. 3b). CFSE labeled NK cells preferentially localized to the liver, as compared to the spleen at 24 h occupying a mean of 0.8% of the total liver lymphocyte population (*p* = 0.03) (Fig. 3c, d). In three out of the seven mice, we also detected NK cells in the tumor, in which they occupied a mean of 1.8% of the tumor-infiltrating lymphocytes in the liver.

To test the concept that these liver localized IL-12/15/18 primed NK cells would have anti-tumor activity we injected c-Myc/TGFα double Tg mice via the tail vein with PBS or with purified NK cells. We performed three infusions of 1 × 10^6^ NK cells 2 weeks apart in the mice aged 12 weeks using littermate controls. Mice were then followed and killed at 24 weeks. Overall, we found that the mean number of tumors was 7.8 in the control mice vs 2.2 in the treated mice (*p* < 0.01). This was associated with a significant decrease in mean liver weight from 4.7 g (untreated controls) vs 3.1 g (treated mice) (*p* < 0.02) (Fig. 3e, f). Thus, these data suggest that cytokine-primed NK cells can localize to the liver and have activity against HCC.

## Discussion

We show that an IL-12/15/18 +IL-2 cytokine cocktail is an effective way to activate human NK cells in vitro and can induce anti-HCC activity. Importantly, NK cells from patients with HCC can be readily activated using this combination of cytokines, suggesting that autologous NK therapy could be possible. However, although in vivo studies have shown that NKG2D ligands are important for HCC outcome, the killing of a panel of HCC cell lines did not correlate well with expression of NKG2D ligands. Further experiments using NKG2D blocking would precisely define the role of NKG2D in killing HCC cell lines. However, the lack of correlation of expression of NKG2D ligands with killing indicates the importance of additional receptor:ligand interactions such as B7-H6 and BAT-3 (the ligands for NKp30), CD155 and CD112 (the ligand for DNAM-1), CD48 [the ligand for CD244 (2B4)] and ICAM-1 [22]. Overall, given the multiplicity of NK cell receptors, perhaps it is not surprising that one single receptor:ligand interaction is not solely responsible for the diversity of killing amongst the tested panel of cell lines.

We have observed some differences using cytokine priming in our work as compared to other investigators. For instance, during in vitro culture Romee et al. observed up-regulation of both CD94 and NKG2A, whereas we observed an unexpected down-regulation of NKp46 [17]. This may be related to our use of IL-2 rather than Il-15 following priming and the fact that we did not remove the IL-12/15/18 following priming. Additionally, using adoptive transfer of murine NK cells, Ni et al. observed no therapeutic effect against subcutaneously injected RMA-S lymphoma cell lines [14]. However, cytokine priming can induce the expression of the liver homing marker CXCR6, and we observed preferential accumulation of NK cells in the liver as opposed to the spleen [23]. Therefore, it may be that the enhanced anti-tumor effect that we observed was related to the liver homing ability of the NK cells. The mechanisms behind the anti-tumor effect of cytokine-primed NK cells require further work, and comparisons with unstimulated NK cells or with NK cells stimulated using different protocols will be required to determine the extent to which this combination of cytokines is necessary for a successful anti-HCC response.

NK cells have been used as therapeutic agents for a number of diseases and are effective against hematological malignancies. Work in solid organ tumors has been less successful [24]. However, HCC provides additional opportunities for NK cell therapies as compared to other cancers. Firstly, tumors can be directly targeted by NK cell infusion during transarterial chemoemblision (TACE). Secondly, the cytokines we tested up-regulate the liver homing marker CXCR6, which may augment targeting of peripherally infused NK cells to the liver [23]. Consistent with this we observed evidence of liver homing during our murine experiments. Thirdly, the ability to generate large number of highly cytotoxic NK cells for infusion through IL-12/15/18 allows an increase in the effector:target ratio of a cell therapy product, which may be advantageous therapeutically.

As we observed killing to similar levels by the NK cells from HCC patients, we would propose that autologous therapy may be possible. Our patient population reflects the patient mix within our clinical practice in that the majority were BCLC stage B. NK cells from individuals with earlier or later stage disease may behave differently. Interestingly, the patients we studied were older than the healthy controls, but retained comparable functions. Furthermore, ageing is thought to affect the NK cell profile within the periphery, but it does not appear to compromise NK cell function [25]. Despite these observations, further characterization of cytokine-primed NK cells derived from individuals with HCC would be valuable in order to understand their precise anti-tumor functionality. Conversely, HLA mis-matching may be beneficial for NK cell therapy against hematological malignancies. Thus, heterologous rather than autologous NK cell therapy may represent an alternative treatment strategy for HCC treatment. However, the c-Myc/TGFα double transgenic mouse has down-regulation of the MHC class I presentation pathway and so we were unable to robustly test this concept [26]. Thus, while we have demonstrated a proof of concept that cytokine-primed NK cells can be useful therapeutically, we have not defined the optimal NK cell therapeutic protocol.

Targeting the TNF-receptor superfamily is one potential therapeutic strategy to enhance NK cell functionality. CD137 (4-1BB) stimulation augments NK cell activation when the agonist is membrane bound, and an agonist antibody can enhance antibody-dependent cellular cytotoxicity [11, 27]. However, previous work has shown that it does not augment natural cytotoxicity and this is consistent with our findings [10]. Additionally, chemotherapeutic agents may be useful in combination with NK cells. This may allow the formation of a niche for NK cell expansion or may augment NK cell activity. For instance, sorafenib, a kinase inhibitor used to treat HCC, can inhibit the shedding of MICA from HCC cells [28] and combinatorial immunotherapy of sorafenib with blockade of programmed death-ligand 1 generates potent immune responses that can result in NK cell-mediated eradication or reduction of tumor growth in tumor-bearing mice [29].

The cytokine-primed NK cells that we have studied have not been used clinically in HCC. However, a recent clinical trial in postoperative HCC using cytokine-induced killer (CIK) cells, which had been activated using a combination of IL-2 and anti-CD3, demonstrated that CIK cells increase both recurrence-free and overall survival [30]. This is an encouraging result, demonstrating the potential for treating HCC with an adoptive immunotherapeutic strategy. However, HCC is a challenging disease to treat as it has a heterogenous etiology, usually arising on the background of cirrhosis, which may be the end result of several different processes including viral infection and metabolic disease. Nevertheless, by having many different activating receptors, NK cells have the potential to target HCC through multiple mechanisms and so may overcome some aspects of this heterogeneity. Consistent with this model, our data demonstrate that IL-12/15/18 activated NK cells have immunotherapeutic potential against HCC in both in vitro cellular experiments and in a murine model of spontaneous HCC. Recent work has shown that NK cells stimulated with this combination of cytokines may be beneficial in refractory acute myeloid leukemia [17]. Therefore, we propose that IL-12/15/18 activated NK cells have potential as an immunotherapy for HCC that warrants further exploration.

## Electronic supplementary material

Below is the link to the electronic supplementary material.

Supplementary material 1 (DOCX 25 kb)

Supplementary material 2 (DOCX 275 kb)
