# Supplementary material for: Activity of IL-12/15/18 primed natural killer cells against hepatocellular carcinoma
Source: Hepatol Int. 2018 Nov 22;13(1):75–83. doi: 10.1007/s12072-018-9909-3 (PMC6513806; doi:10.1007/s12072-018-9909-3)
Supplement: Supplementary file 1 — Supplementary material 1 (DOCX 25 kb) [file 12072_2018_9909_MOESM1_ESM.docx]

**Supplementary Table 1: Data for manuscript figures.**

***Supplementary Table 1a:*** Data for figure 1b

Killing of a panel of liver cancer cell lines by NK cells from six healthy donors. NK cells were either stimulated with cytokines (IL-12/15/18+IL-2) or, stimulated with cytokines and anti-CD137. Shown are the means and standard errors of the percentage of cellular cytotoxicity against the various targets of NK cells stimulated with the different conditions.

|  | **Unstimulated NK** | | **NK+cytokines** | | **NK+cytokines+anti-CD137 Ab** | | |
| --- | --- | --- | --- | --- | --- | --- | --- |
| **Cell line** | Mean | SEM | Mean | SEM | Mean | SEM |  |
| **221** | 9.13 | 3.87 | 66.17 | 2.41 | 54.82 | 2.91 |  |
| **387** | 1.25 | 0.93 | 17.68 | 2.73 | 12.33 | 2.05 |  |
| **398** | 4.05 | 1.30 | 24.50 | 2.92 | 17.72 | 2.51 |  |
| **423** | 0.45 | 0.36 | 10.75 | 3.01 | 5.00 | 1.14 |  |
| **475** | 1.80 | 0.86 | 9.10 | 1.15 | 6.62 | 1.40 |  |
| **Huh7** | 1.05 | 0.95 | 13.40 | 3.19 | 7.38 | 1.62 |  |
| **HepG2** | 2.60 | 2.08 | 45.37 | 8.26 | 33.75 | 5.48 |  |
| **PLC** | 2.15 | 1.14 | 19.90 | 2.91 | 16.00 | 2.28 |  |

***Supplementary Table 1b:*** Data for figure 1c

Expression of markers by NK cells from five healthy donors. NK cells were either stimulated with cytokines (IL-12/15/18+IL-2) or, stimulated with cytokines and anti-CD137. Shown are the means and standard errors of the percentage of expression of the various receptors on CD3-CD56+ NK cells.

|  | **Unstimulated NK** | | **NK+cytokines** | | **NK+cytokines+anti-CD137 Ab** | |
| --- | --- | --- | --- | --- | --- | --- |
| **Marker** | Mean | SEM | Mean | SEM | Mean | SEM |
| **Perforin+** | 94.71 | 1.76 | 91.21 | 2.30 | 87.44 | 3.39 |
| **NKp46+** | 77.48 | 5.05 | 33.59 | 4.44 | 32.21 | 3.42 |
| **NKG2D+** | 36.07 | 3.23 | 68.72 | 4.33 | 69.02 | 5.73 |
| **NKG2A+** | 48.13 | 7.24 | 87.55 | 2.95 | 86.09 | 3.10 |
| **CD158a+** | 37.10 | 4.51 | 28.54 | 4.32 | 31.02 | 4.03 |
| CD158b+ | 33.57 | 5.14 | 32.88 | 4.06 | 34.74 | 3.81 |
| CD137+ | 7.53 | 5.39 | 15.39 | 2.63 | 15.19 | 1.57 |
| CD27+ | 9.44 | 1.10 | 2.94 | 0.68 | 2.95 | 0.67 |

***Supplementary Table 1c:*** Data for figure 1e

Killing of a panel of liver cancer cell lines by NK cells from six healthy donors. NK cells were either unprimed (cultured in IL-2) or primed (cultured overnight in IL-12/15/18, followed by IL-2). Shown are the means and standard errors of the percentage of cytotoxicity against the various targets of unprimed and primed NK cells.

|  | **Unprimed** | | **Primed** | |
| --- | --- | --- | --- | --- |
| **Cell line** | Mean | SEM | Mean | SEM |
| **221** | 21.10 | 13.86 | 74.66 | 5.15 |
| **387** | 9.43 | 2.67 | 8.94 | 3.18 |
| **398** | 8.10 | 9.03 | 22.78 | 8.01 |
| **423** | 6.40 | 4.24 | 22.50 | 2.71 |
| **475** | 6.15 | 1.20 | 11.66 | 5.30 |
| **Huh7** | 25.40 | 0.71 | 31.64 | 14.66 |
| **HepG2** | 26.83 | 6.64 | 36.16 | 12.08 |
| **PLC** | 19.55 | 4.49 | 24.28 | 6.21 |

***Supplementary Table 1d:*** Data for figure 1f

Secretion of IFNγ by NK cells from six healthy donors following culture with a panel of liver cancer cell lines. NK cells were either unprimed (cultured in IL-2) or primed (cultured overnight in IL-12/15/18, followed by IL-2). Shown are the means and standard errors of the percentage of cellular cytotoxicity against the various targets of unprimed and primed NK cells.

|  | **Unprimed** | | **Primed** | |
| --- | --- | --- | --- | --- |
| **Cell line** | Mean | SEM | Mean | SEM |
| **221** | 422.04 | 137.84 | 979.34 | 164.90 |
| **387** | 365.13 | 158.73 | 964.12 | 126.91 |
| **398** | 390.65 | 118.54 | 993.53 | 125.32 |
| **423** | 351.86 | 148.63 | 982.10 | 134.96 |
| **475** | 346.09 | 146.30 | 981.37 | 180.13 |
| **Huh7** | 453.79 | 101.72 | 999.63 | 131.32 |
| **HepG2** | 600.11 | 166.15 | 1014.77 | 74.51 |
| **PLC** | 604.19 | 153.10 | 1023.04 | 112.16 |

***Supplementary Table 1e:*** Data for figure 2b

Proliferation of NK cells from individuals with HCC (n=11) and healthy controls (n=8). Shown are the means and standard errors of the fold increase in cell number of cytokine primed NK cells at various days).

|  | **HCC** | | **Healthy controls** | |
| --- | --- | --- | --- | --- |
| **Day** | mean | SEM | Mean | SEM |
| 3 | 0.77 | 0.17 | 0.91 | 0.23 |
| 5 | 1.27 | 0.32 | 2.18 | 0.27 |
| 7 | 2.37 | 0.36 | 6.94 | 1.24 |
| 9 | 6.08 | 0.35 | 9.75 | 1.43 |

***Supplementary Table 1f:*** Data for figure 2c

Comparison of killing of a panel of liver cancer cell lines by cytokine primed NK cells from HCC patients (n=11) and healthy controls (n=8). Means and standard errors of percentage cytotoxicity are shown at day 9 of culture.

|  | **HCC** | | **Healthy controls** | |
| --- | --- | --- | --- | --- |
| **Cell line** | Mean | SEM | Mean | SEM |
| **221** | 44.43 | 4.90 | 39.66 | 2.36 |
| **387** | 28.47 | 3.47 | 20.71 | 2.74 |
| **398** | 30.40 | 1.97 | 28.06 | 2.00 |
| **423** | 10.00 | 1.64 | 6.34 | 1.09 |
| **475** | 8.51 | 1.58 | 8.66 | 3.09 |
| **Huh7** | 6.53 | 0.46 | 5.49 | 1.45 |
| **HepG2** | 46.47 | 6.44 | 43.66 | 6.71 |
| **PLC** | 28.94 | 3.78 | 25.17 | 4.45 |

***Supplementary Table 1g:*** Data for figure 2e

NKG2D expression on cytokine primed CD3-CD56+NK cells from twelve HCC patients and seven healthy controls at day 9. Shown are the mean percentage positive NK cells and the standard errors of the means.

|  | **HCC** | | **Healthy controls** | |
| --- | --- | --- | --- | --- |
|  | Mean | SEM | Mean | SEM |
| **Day 0** | 56.12 | 3.23 | 55.19 | 3.79 |
| **Day 9** | 93.62 | 1.55 | 95.53 | 0.79 |
|  |  |  |  |  |

***Supplementary Table 1h:*** Data for figure 2f

Receptor expression on cytokine primed CD3-Cd56+ NK cells from twelve HCC patients and seven healthy controls at day 9. Shown are the mean percentage positive NK cells and the standard errors of the means.

|  | **HCC** | | **Healthy controls** | |
| --- | --- | --- | --- | --- |
| **Marker** | Mean | SEM | Mean | SEM |
| **NKp46** | 39.33 | 3.59 | 43.25 | 4.23 |
| **NKG2A** | 86.23 | 2.99 | 86.21 | 2.92 |
| **CD158a** | 16.22 | 2.77 | 18.31 | 2.69 |
| **CD158b** | 25.25 | 2.81 | 20.85 | 2.65 |
| **CD137** | 48.93 | 4.22 | 49.78 | 4.51 |
| **CD27** | 3.23 | 0.19 | 3.61 | 0.29 |
