# Supplementary material for: Activity of IL-12/15/18 primed natural killer cells against hepatocellular carcinoma
Source: Hepatol Int. 2018 Nov 22;13(1):75–83. doi: 10.1007/s12072-018-9909-3 (PMC6513806; doi:10.1007/s12072-018-9909-3)
Supplement: Supplementary file 2 — Supplementary material 2 (DOCX 275 kb) [file 12072_2018_9909_MOESM2_ESM.docx]

**Supplementary Figures**

**Supplementary Figure 1**

Flow cytometry plots illustrating post-purification purity of NK cells from one human donor (right panel) and one murine spleen (left panel)

**Supplementary Figure 2**

Flow cytometry plots illustrating a representative NK cell proliferation assay from a single healthy donor
